# First-Line Therapy for Human Cutaneous Leishmaniasis in Peru Using the TLR7 Agonist Imiquimod in Combination with Pentavalent Antimony

**DOI:** 10.1371/journal.pntd.0000491

**Published:** 2009-07-28

**Authors:** Cesar Miranda-Verastegui, GianFranco Tulliano, Theresa W. Gyorkos, Wessmark Calderon, Elham Rahme, Brian Ward, Maria Cruz, Alejandro Llanos-Cuentas, Greg Matlashewski

**Affiliations:** 1 Universidad Peruana Cayetano Heredia, Lima, Peru; 2 Division of Clinical Epidemiology, Research Institute of the McGill University Health Centre, Montreal, Canada; 3 Social Security Hospital ESSALUD-Cusco, Lima, Peru; 4 Department of Microbiology and Immunology, McGill University, Montreal, Canada; Institute of Tropical Medicine, Belgium

## Abstract

**Background:**

Current therapies for cutaneous leishmaniasis are limited by poor efficacy, long-term course of treatment, and the development of resistance. We evaluated if pentavalent antimony (an anti-parasitic drug) combined with imiquimod (an immunomodulator) was more effective than pentavalent antimony alone in patients who had not previously been treated.

**Methods:**

A randomized double-blind clinical trial involving 80 cutaneous leishmaniasis patients was conducted in Peru. The study subjects were recruited in Lima and Cusco (20 experimental and 20 control subjects at each site). Experimental arm: Standard dose of pentavalent antimony plus 5% imiquimod cream applied to each lesion three times per week for 20 days. Control arm: Standard dose of pentavalent antimony plus placebo (vehicle cream) applied as above. The primary outcome was cure defined as complete re-epithelization with no inflammation assessed during the 12 months post-treatment period.

**Results:**

Of the 80 subjects enrolled, 75 completed the study. The overall cure rate at the 12-month follow-up for the intention-to-treat analysis was 75% (30/40) in the experimental arm and 58% (23/40) in the control arm (p = 0.098). Subgroup analyses suggested that combination treatment benefits were most often observed at the Cusco site, where *L. braziliensis* is the prevalent species. Over the study period, only one adverse event (rash) was recorded, in the experimental arm.

**Conclusion:**

The combination treatment of imiquimod plus pentavalent antimony performed better than placebo plus pentavalent antimony, but the difference was not statistically significant.

**Trial Registration:**

Clinical Trials.gov NCT00257530

## Author Summary

Neglected tropical diseases (NTDs) are a group of tropical infections including trypanosomiasis, filariasis, schistosomiasis, onchocerciasis, leishmaniasis and other such diseases of poverty. Of the classic neglected diseases, leishmaniasis has among the highest level of morbidity and mortality. Infection with *Leishmania* parasites causes severe disease in humans, including fatal visceral leishmaniasis and cutaneous leishmaniasis resulting in severe scarring, often in the face. This is a difficult infection to treat because the current therapies are generally poorly effective. The present study carried out a placebo-controlled, double-blinded study to investigated whether a combined therapy with imiquimod plus pentavalent antimony was superior to the standard therapy of pentavalent antimony alone as a first-line treatment for cutaneous leishmaniasis in Peru. A higher cure rate with the combination therapy was observed, but could not be conclusively proven.

## Introduction

Leishmaniasis includes a spectrum of diseases occurring throughout Asia, Africa, and the Americas which are caused by infection with *Leishmania* parasites transmitted by the bite of infected sandflies [1]. Disease manifestations are determined predominantly by the host's immune response and the parasite species [2]. In Peru, the predominant species include the *Leishmania (Viannia)* complexes of *L. braziliensis*, *L. peruviana* and *L. guyanensis* that are all associated with cutaneous leishmaniasis. Mucocutaneous leishmaniasis is caused predominantly by *L. braziliensis* infection [3],[4].

There is no vaccine for leishmaniasis and current therapies are limited by poor efficacy, the requirement for prolonged treatment, and increasing development of clinical resistance. The drugs most commonly used include pentavalent antimonials, various amphotericin B lipid formulations and a variety of other drugs used to a lesser extent, including pentamidine, miltefosine, and paromomycin [5]. In Peru, the most commonly used first-line treatment for cutaneous and mucocutaneous leishmaniasis is pentavalent antimony (meglumine antimoniate or sodium stibogluconate) with a success rate varying between 60% and 80% [6]. Amphotercin B is typically used in those patients who do not respond to pentavalent antimony. The current standard treatment regimes for cutaneous leishmaniasis all involve monotherapy. The use of combination therapy may improve efficacy, and if toxic drugs can be used at lower levels, improve tolerance.

Host immune mechanisms play an important role in the efficacy of anti-*Leishmania* chemotherapy [reviewed in 7]. An essential component of cell-mediated immunity against *Leishmania* is the development of a Th1 type response that activates macrophages via IFN-γ to either inhibit or kill the parasite [2]. Activation of the innate immune response is essential for the subsequent development of the Th1 type cell-mediated immune response. Imiquimod is a small molecule that activates Toll-like receptors 7 and 8 (TLR 7/8) on antigen-presenting cells and mediates the production of a variety of cytokines including IFN-α, IFN-γ, TNF-α, IL-1 and IL-12 leading to the induction of enhanced Th1 immune responses [8],[9]. In addition, it has been demonstrated that imiquimod can directly activate macrophage killing of *Leishmania* amastigotes in the absence of a T-cell-mediated response [10]. Enhancing the local immune response at the site of cutaneous infection, therefore, may be a logical approach to enhance parasite clearance.

We previously reported that combination therapy with imiquimod plus parental pentavalent antimony was more effective than pentavalent antimony alone in patients who had previously failed treatment with pentavalent antimony [11]. The objective of this clinical trial was to determine whether the combination therapy can also be beneficial for cutaneous leishmaniasis patients who have not previously been treated.

## Methods

### Trial Design

The study design was a randomized controlled trial where 80 subjects were recruited at two clinics associated with the leishmaniasis clinic at the Instituto de Medicina Tropical ‘Alexander von Humbolt’ - Universidad Peruana Cayetano Heredia (UPCH) – Hospital Nacional Cayetano Heredia (in Lima and in Cusco, Peru). Subjects recruited in Lima are typically infected with *L. peruviana*, *L. guyanensis*, or *L. braziliensis* while those recruited at the Cusco site are infected predominantly with *L. braziliensis* [12]. We therefore planned to analyze the data overall and for the Lima and Cusco sites individually. Each clinic recruited 20 experimental and 20 control subjects and assigned treatment based on a 1∶1 randomization list generated by 3M Pharmaceuticals Inc. Subjects assigned to the control arm received the standard pentavalent antimony treatment plus an application of placebo vehicle cream applied to each lesion 3 times per week. Subjects assigned to the experimental arm received pentavalent antimony plus 5% imiquimod cream identically applied. Study I.D. numbers and corresponding treatment packages were prepared so that both subjects and study investigators were blind to treatment allocation throughout the study. Although diagnostic and clinical procedures were performed in either Lima or Cusco, most subjects were recruited from smaller communities of within a one day's drive (e.g. Ancash, Churin, Yumpe, Cajatambo, Yauyos, Sicuani, Madre de Dios and surrounding areas). Study teams at each clinic comprised doctors, nurses, and technologists, all of whom were supervised by Dr. Llanos-Cuentas. Following the completion of therapy, follow-up visits were scheduled at 1, 2, 3, 6, 9 and 12 months. The primary outcome was cure, defined as complete re-epithelization with no inflammation.

### Eligibility Criteria

The following inclusion and exclusion criteria were assessed using a pre-screening protocol to determine patients' eligibility.

Inclusion criteria:

- Males and females between 5 and 65 years of age
- Confirmed diagnosis of cutaneous leishmaniasis (i.e. presence of an active ulcerative cutaneous *Leishmania* lesion, and a positive identification of the parasite from the lesion. (smear microscopy, culture, or PCR)
- Duration of disease more than 4 weeks.
- No prior therapy with anti-*Leishmania* drugs.
- Female patients of childbearing age: a negative urine pregnancy test, not breastfeeding, required to use adequate contraception during the 20-day treatment.
- Informed written consent (self or parent for under-18 year-olds) for the trial and a separate additional consent for photos of lesions (at baseline and at follow-up time points).
- Willing to participate in all treatment and follow-up visits, and be reachable by study personnel.

Exclusion criteria:

- Lesion(s)>2,500 mm^2^.
- More than 6 cutaneous lesions.
- Mucosal lesion.
- Previous exposure to imiquimod or anti-*Leishmania* treatment.
- Participation in another experimental protocol and/or had received investigational products within previous 30 days.
- History of any acute or chronic illness (other than cutaneous leishmaniasis) or medication that, in the opinion of the investigators, may interfere with the evaluation of the trial (e.g. history of heart or liver illness).
- History of significant psychiatric illness.
- History of previous anaphylaxis or severe allergic reaction to one or more of the proposed drugs.
- Unlikely to cooperate with the requirements of the study protocol.
- Concomitant infection (i.e. bartonellosis, sporotrichosis, mycobacterial infection)

Before enrollment, subjects also underwent an electrocardiogram and blood sampling for limited biochemical analysis (eg. ALT, AST, total bilirubin, alkaline phosphatase, pancreatic amylase, glucose and creatinine). When a bacterial superinfection was suspected at the site of *Leishmania* infection, subjects received oral or systemic antibiotic treatment (dicloxacillin or clindamycin) before entry into the trial (during the screening period).

### Baseline Measurements

Subjects who met all inclusion/exclusion criteria underwent a limited physical exam to ensure that no mucosal lesion had developed. Borders around each lesion were drawn using a plastic sheet placed on the lesion to accurately document size. Lesions were photographed with a digital camera (at daylight setting (no flash) at a distance of 15 cm, with patient ID code and date clearly indicated beside the millimeter ruler. The photos were stored in a computer at UPCH in Lima. A punch biopsy specimen was taken from the border of the lesion prior to the first treatment and a section placed into media to culture the parasite.

### Treatment

#### Antimonial treatment

All subjects received pentavalent antimony. The dose of 20 mg sodium stibogluconate (Albert David, Kolkata, India) per kg body weight/day was administrated intravenously for 20 consecutive days. Because a total of 20 injections were administered, care was taken to ensure that injections were not administered in the same exact location.

#### Imiquimod and placebo cream treatments

The treatment sachets containing the assigned topical cream for each patient were opened at the time of each treatment and the imiquimod or placebo vehicle cream (3M Pharmaceuticals Inc.) applied to the cutaneous lesion. Both creams were identical in appearance. At each treatment, the study nurse cleaned the lesion using sterile saline solution to remove debris or scab and then applied the cream on the entire area of each lesion including a 0.5 cm margin of normal skin, using a gloved finger. The cream was applied in the morning and rubbed into the lesion(s) until no longer visible. Each sachet contained 250 mg of cream (with or without active imiquimod) and each lesion received between 125–250 mg of cream. Topical treatment was applied three times per week (i.e. Monday, Wednesday, Friday or Tuesday, Thursday and Saturday) for a total of 9 applications during the 20-day course of treatment with pentavalent antimony. Only new unopened sachets were used for each treatment.

The lesion(s) remained uncovered for at least 30 minutes following each application during which time the study doctor recorded all symptoms of the patient. All lesions except those on the face were covered with an occlusive dressing (Tegaderm, 3M Pharmaceuticals Inc.) to prevent cream removal by clothing. The patients were instructed to remove the dressing and wash the lesions with warm soapy water approximately 8 h following treatment, as well as each morning. After each treatment, the study doctor evaluated the patient for 30 minutes and the patient was encouraged to continue his/her daily activities.

### Evaluation of Clinical Response

The primary outcome was cure defined as complete re-epithelialization with no inflammation assessed at between 1 and 12 months post-treatment. The clinical response was evaluated using standard criteria for assessing the size and severity of the cutaneous lesion including size of lesion, reduced ulceration, re-epithelialization, and inflammation reduction. The criteria used to evaluate the response to treatment have been used for the past several years by the investigators [7],[8] as follows.

**M0:** No improvement. The lesion is asctive and has the same characteristics of has become larger than before the start of treatment.
**MI:** The size of the lesion is decreased ∼50% in comparison with the initial lesion. Less inflammatory signs with discrete re-epithelialization.
**M2:** The size of the lesion is decreased between 50–90% in comparison with the initial lesion. Few inflammatory signs, less than M1.
**M3:** The size of the lesion is decreased more than 90% with re-epithelialization and very little inflammation.
**M4:** Complete re-epithelialization with a characteristic scar and no inflammation.

At each of the follow-up time points (ie. at 1, 2, 3, 6, 9, 12 months), the overall response to therapy was classified as: Improvement, Cure or Failure based on the characteristics listed below:

Improvement: Significant reduction in the size of the lesion at the time of evaluation (Stages M1–M3) compared to baseline.

Cure: Complete re-epithelialization of the lesion without inflammation (Stage M4).

Failure: Any one, or combination, of the following options:

i) Stage MO at 1 month of follow-up after the treatment
ii) Stage M1 at 3 months of follow-up after the treatment
iii) Regression of the clinical stage to a lower stage or development of a new lesion.

### Safety and Tolerability Evaluation

Local side effects of the topical treatment at the application site (pain, pruritus, erythema and swelling) were graded as follows: Grade 0 = none; Grade 1 = mild (easily tolerated); Grade 2 = moderate (sufficiently discomforting to interfere with daily activities); Grade 3 = severe (prevents normal daily activity).

### Follow-Up Evaluations

At each follow-up visit after the preceding treatment, a lesion evolution score was assigned based on the least improved lesion in the case of patients with more than one lesion and was recorded in the clinical report form (CRF). This evaluation included measuring the size of the lesion(s) and taking a standardized photograph of each lesion. A limited physical examination was also performed, recording all adverse events experienced since the last follow-up visit.

### Parasite Diagnosis and Species Identification

The clinical diagnosis of cutaneous leishmaniasis was confirmed in all patients by directly identifying the parasite by smear (Giemsa staining), by culture (in Novy-Macneal Nicolle media), and/or by PCR of the *Leishmania* minicircle DNA prior to randomization. *Leishmania* species determination was possible in those cases where the parasite was successfully cultured from the lesion. To differentiate between infections with *L. braziliensis*, *L. peruviana*, and *L. guyanensis*, the mannose phosphatase isomerase isoenzyme (MPI) gene was sequenced from the cultured parasites. The MPI enzyme represents the only isoenzyme identified by Multi Locus Enzyme Electrophoresis (MLEE) to differentiate these species [12] and it has recently been established that the sequence of the MPI gene could differentiate between infections with these different species [13],[14]. All parasite species determinations were performed before the treatment blind was broken.

### Statistical Analysis

Sample size was estimated such that the log-rank test for equality of survival curves would have 80% power to detect a statistically significant difference in proportions cured at three months of at least 32% (hazard ratio of 2.6) (estimates of proportions cured at 3 months were based on previously published data) [11]. This test assumes a constant hazard ratio over time.

Descriptive statistics (means±standard deviations (SD) and proportions) were calculated to summarize socio-demographic, clinical and epidemiological characteristics of patients at baseline for experimental and control arms. These characteristics included age, sex, study site, region of infection acquisition, occupation and lesion-specific data (number, location, size, type, duration, presence of adenopathy and presence of bacterial superinfection).

Intention-to-treat and efficacy analyses were conducted. The intention-to-treat analysis was the primary analysis. In this analysis, missing values at day 20 were treated as failures and any subsequent missing value was assigned the outcome status of the patient's most recent visit. The effect of this decision in dealing with missing values, was examined in a secondary intent-to-treat analysis where all non-cured categories (missing and not improved/worse) were considered failures. Additional secondary analyses were performed. This included an efficacy (per protocol) approach and a comparison of the proportions of subjects cured at each timepoint using chi-square tests. The log rank test was used to compare the Kaplan-Meier survival curves.

### Ethics Review Board Approval and Informed Consent

The experimental protocol for this study was designed in accordance with the general ethical principles outlined in the Declaration of Helsinki, 2000 and ICH (International Committee for Harmonization) guidelines for Good Clinical Practice. Approvals from the ethics review boards of Universidad Peruana Cayetano Heredia UPCH, McGill University, and the National Institute of Health in Peru (INS-Peru) were obtained prior to the initiation of the study. The UPCH and INS review committees approved a Spanish version of the consent form. The study investigators explained the nature of the investigation and the risks involved to each patient, or parent of a patient below 18 years of age, prior to recruitment. Written informed consent was obtained from each patient enrolled in the study and the patient/parent was informed that he/she was free to voluntarily withdraw from the study at any time.

### Study Monitoring

Program directors from DNDi and a study monitor employed by DNDi periodically reviewed study documentation (ie. all CRFs and SOPs and corresponding source documents for each patient) and implementation procedures. The monitoring visits provided DNDi with the opportunity to evaluate the progress of the study, verify the accuracy and completeness of CRFs, resolve any inconsistencies in the study records, as well as to ensure that all protocol requirements, applicable regulations, and investigators' obligations were fulfilled.

### Statement on Conflict of Interest

There was no conflict of interest in this study. 3M Pharmaceuticals Inc. provided the randomized allocation of imiquimod and placebo creams at no cost; however, they did not provide financial support for this study, nor were they otherwise involved in study design, data analysis, interpretation of the data or in the writing of the manuscript.

## Results

**Figure 1:**
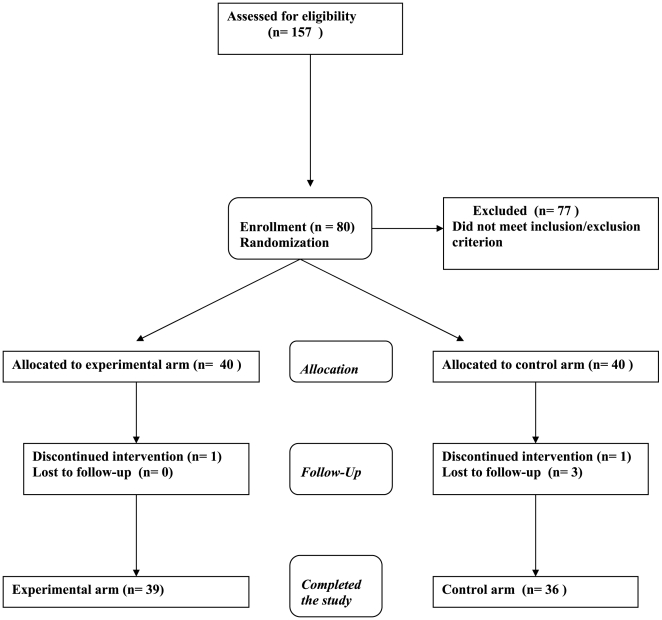
Trial flowchart.

Patient recruitment took place between December 2005 and June 2006; follow-up continued to June 2007. From the 157 patients screened, 80 patients met the inclusion/exclusion criteria, 78 (97.5%) completed the 20-day course of treatment, and 75 (93.8%) were followed for the entire 12 months following the end of therapy (Figure 1). The demographic characteristics of the patients are presented in Table 1. There were no significant differences between the intervention groups.

**Table 1:** Baseline demographic characteristics of subjects with leishmaniasis treated with imiquimod 5% cream plus pentavalent antimony versus placebo cream plus pentavalent antimony, participating in a randomized trial, Peru, 2006–7.

Characteristic	Imiquimod plus pentavalent antimony	Placebo plus pentavalent antimony
	(*n* = 40)	(*n* = 40)
Age (in years)		
Mean±SD	25.0±10.3	25.9±10.4
Range	(6 to 52)	(4 to 52)
Sex (no. of subjects)		
Male	31	31
Female	9	9
Study site (no. of subjects)		
Lima	20	20
Cusco	20	20
Occupation (no. of subjects)		
Agriculture	22	26
Professional	2	1
Mining	4	4
Student	2	1
Tourism	6	7
Other	4	1
Region where leishmaniasis was acquired (no. of subjects)^a^		
Mountains	5	9
Jungle	35	31
No. subjects who completed 20-day combination therapy with pentavalent antimony plus the imiquimod or placebo cream	39	39

a Mountains include the Andes between 1000 and 2500 meters above sea level; Jungle includes the Amazonian forest between 0 and 1000 meters above sea level.

The principal characteristics of the lesions are presented in Table 2. On average, each patient had 2 lesions. The mean duration and sizes of the lesions were not judged to be different between the experimental: antimony/imiquimod and control: antimony/placebo groups. There were more lesions on the torso in the control: antimony/placebo group (n = 14 lesions in 5 subjects) than in the experimental: antimony/imiquimod group (n = 0). There were more lesions with bacterial super-infections in the experimental: antimony/imiquimod group (n = 9 lesions in 4 subjects) than in the control: antimony/placebo group (n = 1). There is no evidence to suggest that these differences could have influenced the outcome of the trial.

**Table 2:** Baseline characteristics of lesions in subjects with leishmaniasis treated with imiquimod 5% cream plus pentavalent antimony versus placebo plus pentavalent antimony, participating in a randomized trial, Peru, 2006–7.

Characteristic	Imiquimod plus pentavalent antimony	Placebo plus pentavalent antimony
	(*n* = 40)	(*n* = 40)
Number of lesions	79	80
Mean±SD	1.98±1.37	2.00±1.47
Median (range)	1 (1 to 6)	1 (1 to 6)
Location of lesion, no. of lesions		
Face	12	13
Upper extremity	23	19
Lower extremity	41	33
Torso^*^	0	14
Pelvis	3	1
Duration of the disease, months		
Mean±SD	2.84±2.20	3.50±3.32
Median (range)	2 (.1 to 10.0)	2 (.1 to 15.0)
Area of lesions (total area per lesion), mm^2^		
Mean±SD	146.24±270.50	178.34±278.75
Median (range)	66 (0 to 1913)	61 (0 to 1554)
Type of lesion, no. of lesions		
Ulcer	78	74
Nodule	1	4
Verrucose	0	1
Scar	0	1
Adenopathy, no. of lesions		
Present	29	25
Absent	50	55
Bacterial superinfection, no. of lesions#		
Present	9	1
Absent	70	79

There were no significant differences between the intervention groups with respect to adverse events at the site of application (Table 3). One patient in the experimental antimony/imiquimod group however had a serious adverse event and was unable to complete the full course of treatment. This was a 39-year-old male who developed a generalized rash on day 9 of treatment, at which time treatment was suspended and he was treated with anti-histamines. After the rash subsided, treatment resumed on day 13 but was discontinued the next day when the rash returned and again treated with anti-histamines to resolve the rash. He was then given only imiquimod cream for the remainder of the 20 days without recurrence of the rash. The rash was considered to be due to the pentavalent antimony. At the 2-month follow-up period, this patient was considered a treatment failure and was given amphotericin B, which resulted in a complete cure with no rash. He was considered to be a treatment failure in the final analysis.

**Table 3:** Adverse effects experienced by subjects with leishmaniasis treated with imiquimod 5% cream plus pentavalent antimony versus placebo cream plus pentavalent antimony, participating in a randomized trial, Peru, 2006–7.

Adverse effect	Imiquimod plus pentavalent antimony	Placebo plus pentavalent antimony	P-value
	(*n* = 40)	(*n* = 40)	
Adverse effect			0.654
Any	20	18	
None	20	22	
Swelling			0.617
Yes	12	10	
No	28	30	
Itching			0.793
Yes	10	9	
No	30	31	
Pain			0.745
Yes	5	6	
No	35	34	
Erythema			0.626
Yes	13	11	
No	27	29	

The evolution of the lesions by intervention group is detailed in Table 4. Most missing values occurred at month 2 (n = 28), but at the 12-month evaluation, outcome data were obtained from 75 of the 80 patients. In the primary intention-to-treat analysis (Table 5), the overall cure rate was 75% (30/40) in the experimental: antimony/imiquimod group compared to 58% (23/40) in the control: antimony/placebo group (p = 0.098) at 12 months. In the secondary intention-to-treat analysis (Table 4), where all non-cured categories (missing, not improved/worse) were considered failures, a statistically significant difference was found in cure rates between the two groups at 12 months.

**Table 4:** Response of lesions to treatment among subjects treated with imiquimod 5% cream plus pentavalent antimony versus placebo cream plus pentavalent antimony, intention-to-treat analysis (missing = failures).

Time after the end of therapy, response	No. (%) of subjects		*P-value* 1
	Imiquimod plus pentavalent antimony	Placebo plus pentavalent antimony	
	(N = 40)	(N = 40)	
At day 20			
Missing*	1	1	
Not improved/worse^a^	0	0	
Improved^b^	37	32	
Cured^c^	2	7	
At month 1			
Missing*	6	5	
Not improved/worse	1	4	
Improved	16	18	
Cured	17 (43%)	13 (33%)	.488
At month 2			
Missing*	10	18	
Not improved/worse	3	5	
Improved	3	5	
Cured	24 (60%)	12 (30%)	
At month 3			
Missing*	1	3	
Not improved/worse	8	13	
Cured	31 (78%)	24 (60%)	0.148
At month 6			
Missing*	1	2	
Not improved/worse	9	13	
Cured	30 (75%)	25 (63%)	
At 9 months			
Missing*	1	4	
Not improved/worse	9	13	
Cured	30 (75%)	23 (58%)	
At month 12			
Missing*	1	4	
Not improved/worse^c^	9	15	
Cured	30 (75%)	21 (53%)	0.036

*Missing includes, at day 20, subjects who did not complete treatment; for all other timepoints, missing also includes those not observed and those lost-to-follow-up.

a Not improved/worse is considered a treatment failure and was defined at 1 month post-treatment as an active lesion, unchanged or worse, compared with its characteristics at the start of treatment; at 3 months post- treatment, as a lesion that has been reduced in size by not more than approximately 50%, although having less inflammatory signs with discrete re-epithelialization compared to the start of treatment; at 6 months and later time points, as a lesion that had regressed to a lower clinical stage or the development of a new lesion.

b Improved is defined as significant reduction in the size of the lesion at the time of evaluation compared to baseline.

c Cured is defined as complete re-epithelialization of the lesion without inflammation.

1 p-values are based on the chi-square analysis comparing cure rates between the two intervention groups, where all those missing and those not improved/worse are considered as failures.

We further performed an efficacy analysis where the patients who did not complete the treatment (one from each group) and those who were lost during the follow-up (three from the placebo group) were not considered in the analysis. In this analysis, the cure rate at the final 12-month follow-up was 77% (30/39) in the experimental antimony/imiquimod group and 58% (21/36) in the control antimony/placebo group (p = 0.085).

**Figure 2:**
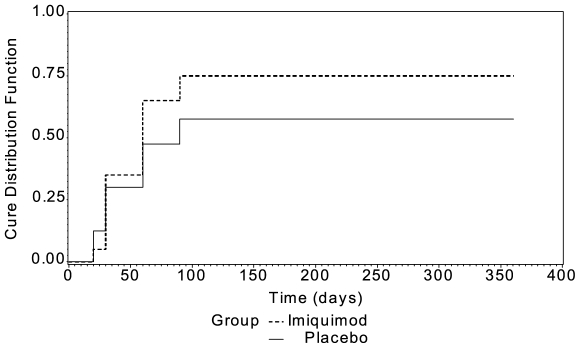
Survival curves comparing imiquimod 5% cream plus pentavalent antimony (dashed line) versus placebo cream plus pentavalent antimony (solid line) in a randomized trial, Peru, 2006–2007. Note: Relapses (n = 3) were considered failures throughout since once they are cured they could not be brought back into the Kaplan-Meier analysis. Missing at day 20 were considered failures (n = 2); for all other timepoints, missing were considered to have the outcome status of their most recent visit.

Figure 2 displays the Kaplan-Meier (KM) curves depicting time to cure for each group within the 12-month period following enrolment into the study. Relapses (n = 3) were considered failures throughout. Missings were considered as described for Table 5. The log rank test comparing the KM curves of the two groups revealed a non- statistically significant result (p = 0.174), although the combined therapy group demonstrated a higher probability of cure compared to the placebo group.

**Table 5:** Response of lesions to treatment among subjects treated with imiquimod 5% cream plus pentavalent antimony versus placebo cream plus pentavalent antimony, intention-to-treat analysis (missing = last value carried forward).

Number cured at each timepoint (%)	No. (%) of subjects		*P-value* 1
	Imiquimod plus pentavalent antimony	Placebo plus pentavalent antimony	
	(N = 40)	(N = 40)	
Day 20	2 (5%)	7 (18%)	0.077
Month 1	17 (43%)	14 (35%)	0.491
Month 2	28 (70%)	21 (53%)	0.108
Month 3	31 (78%)	24 (60%)	0.091
Month 6	30 (75%)	24 (60%)	0.152
Month 9	30 (75%)	25 (63%)	0.227
Month 12	30 (75%)	23 (58%)	0.098

*: Missing at day 20 are considered failures; for all other timepoints, missing are considered to have the outcome status of their most recent visit.

1 p-values are based on the Pearson chi-square analysis comparing cure rates between the two intervention groups.

Patients who did not respond to therapy after 2–3 months follow-up were treated with amphotericin B, and all infected lesions were cured. All of these subjects were considered as treatment failures in the intention-to-treat analyses.

Analysis by treatment center (intention-to-treat, primary analysis) revealed that, at 12 months, the Lima cohort had a 75% cure rate in the experimental: antimony/imiquimod group compared to 65% in the control: antimony/placebo group (p = 0.490) (Table 6). In comparison, the Cusco cohort had a 75% cumulative cure rate in the experimental: antimony/imiquimod group compared to a 50% cure rate in the control: antimony/placebo group (p = 0.102). Efficacy analysis for the Cusco cohort, removing patients who did not complete therapy or were lost to follow-up, showed a cure rate in the experimental: antimony/imiquimod group of 79% (15/19) compared to 47% (8/17) in the control: antimony/placebo group (p = 0.046).

**Table 6:** Cure rate at the 12-month follow-up time point: By site.

	pentavalent antimony plus imiquimod	pentavalent antimony plus placebo	
Patient status at 12 months: Intention-to-treat analysis			
OVERALL			
Number of patients	40	40	80
Cured	30	23	
Failed	10	17	
LIMA			
Number of patients	20	20	40
Cured	15 (75%)	13 (65%)	
Failed	5	7	X^2^ = 0.476; p = 0.490
CUSCO			
Number of patients	20	20	40
Cured	15 (75%)	10 (50%)	
Failed	5	10	X^2^ = 2.667; p = 0.102

*: Missing at day 20 are considered failures; for all other timepoints, missing are considered to have the outcome status of their most recent visit.

1 p-values are based on the chi-square analysis comparing cure rates between the two intervention groups.

**Figure 3:**
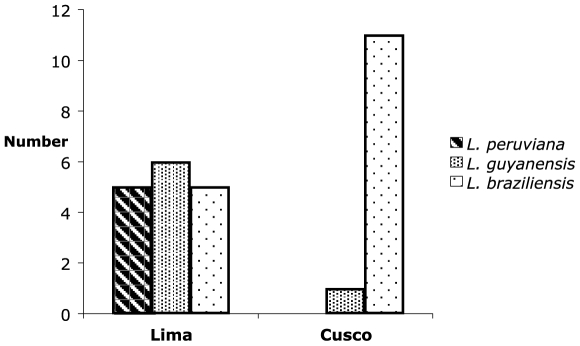
Distribution of the 28 speciated *Leishmania* infections, by site.

The major difference between treatment center appeared to be the lower cure rate in the Cusco control: antimony/placebo group. Baseline characteristics of the lesions from the patients from Lima and Cusco are shown in Table 7 and show that the lesions were, on average, larger in the Cusco cohort. *L. braziliensis* infections are generally more difficult to cure and have a higher relapse rate than *L. peruviana* and *L. guyanensis* infections and could account for the lower cure rate in the Cusco control: antimony/placebo group (6, 15). Species determination, performed as previously detailed [14],[15] on 28 cultures established from patient lesions revealed that *L. braziliensis* infections were present in 11 of 12 subjects from Cusco and 5 of 16 from Lima (Figure 3). The distribution of infecting species was consistent with prior studies showing that patients in Cusco are predominantly infected with *L. braziliensis* [12],[16] and this could also explain why the lesions from the Cusco cohort were on average larger than the lesions from the Lima cohort.

**Table 7:** Baseline characteristics of subjects with leishmaniasis, by study site (Lima and Cusco).

	LIMA	CUSCO
Age (mean in years)	24.6	26.3
Sex (% male)	68	88
Duration (mean in months)	3.45	3.10
Number of lesions per patient (mean)	2.03	1.93
Size of lesions (mean in mm^2^)	119.4	213.6

## Discussion

This study assessed whether the combination of topical imiquimod plus systemic pentavalent antimony was superior to monotherapy with pentavalent antimony as a first-line treatment for cutaneous leishmaniasis in Peru. The final outcome did not reach statistical significance in the intention-to-treat analysis, although the combination therapy did have a higher cure rate. In the planned subset analysis by site, there was a similar suggestion that the combination therapy was superior to monotherapy in the Cusco cohort which was predominantly infected with *L. braziliensis*, a species usually more difficult to treat than *L. peruviana* and *L. guyanensis* [6]. The results from this trial are consistent with a previous open-label pilot trial involving this combination therapy as a first-line therapy for cutaneous leishmaniasis in Peru [17] as well as trials on patients who had previously failed treatment [11],[18].

An important consideration of this study is that activation of a toll like receptor (TLR) is therapeutically beneficial in the treatment of this parasitic infection and this could have implications beyond leishmaniasis. Activation of TLR 7/8 with imiquimod could work in at least two ways to enhance resolution of *Leishmania* infections. First, it could directly stimulate macrophages to synthesize nitric oxide resulting in direct killing of the parasite, as previously detailed in *Leishmania*-infected cultured macrophages [10]. Secondly, imiquimod could mediate a better anti-*Leishmania* Th1 immune response that would result in the production of IFN-γ and macrophage activation resulting in enhanced parasite killing. This would be consistent with the observation that topical imiquimod, used as a vaccine adjuvant with a killed *Leishmania* preparation, has been shown to enhance the Th1 immune response against a subsequent live infection with *L. major* in BALB/c mice [19]. Since the immune status of leishmaniasis patients is known to affect drug efficacy [7], the parasite killing by pentavalent antimony plus the enhanced innate and/or acquired immune response against *Leishmania* could be expected to act synergistically to increase the cure rate and/or reduce the relapse rate.

We have previously observed that treatment with imiquimod alone had a transient beneficial effect as determined by a reduction in lesion size in the first few days of treatment but did not ultimately clear the infection [17]. This has also been observed for Old World cutaneous leishmaniasis [20]. This could suggest that imiquimod can activate an initial innate immune response as previously observed in infected and non-infected macrophages *in vitro* [10],[21]. Future studies should include analyses of relevant immunological parameters in the infected lesions treated with topical imiquimod to provide a better understanding of how imiquimod enhances cure and possibly define more effective therapy regimes.

An interesting observation was that there was a higher failure rate in the control: antimony/placebo group in the Cusco cohort compared to the Lima cohort. This could be related to the predominance of *L. braziliensis* in Cusco as observed in this and previous studies [12],[16]. As there is a higher failure and/or relapse rate with *L. braziliensis* infections than with *L. peruviana* or *L. guyanensis* infections [6],[15], it is possible that the combination therapy may have reduced the relapse rate associated with *L. braziliensis* infections in the Cusco cohort. However, in addition to the different species, there may be other differences between the two sites that necessitate a larger sample size. In any event, if the combination therapy were to be considered for *L. braziliensis* infections, it would be necessary to implement a pre-treatment species determination.

Two previous studies using the combination of imiquimod with pentavalent antimony have been carried out in Iran in areas endemic for *L. tropica*. One study reported that there was no beneficial effect [22] and the other study reported a beneficial effect of the combination therapy [23]. It is difficult to compare our Peruvian study with these results as there were differences in: definitions for cure, methods of administration of the pentavalent antimony, length of follow-up, and prevalent *Leishmania* species. It is noteworthy that, in Iran, *L. tropica* is resistant to pentavalent antimony treatment [24] and this could further complicate the interpretation of the outcome. It would, however, be of interest to carry out an imiquimod combination immunotherapy trial in the Old World involving *L. major* infections that are responsive to pentavalent antimony or in combination with other anti-*Leishmania* treatments.

There was one adverse event that resulted in deviation from the treatment protocol when a generalized skin rash developed in one patient in the experimental (antimony/imiquimod) group. The rash resolved when the pentavalent antimony treatment was stopped and the patient continued to be treated with topical imiquimod alone. This patient was eventually considered a treatment failure at 2 months follow-up and was counted as a failure in the experimental group even though he had not received the complete course of combination therapy.

Future studies should include combining topical imiquimod with other therapies for cutaneous leishmaniasis such as, for example, with topical paromomycin or oral miltefosine. If the cure rate with a combined imiquimod/paromomycin or imiquimod/miltefosine therapy reaches a cure rate of 80% at 3 months post-treatment, then this can be used to replace pentavalent antimony as the first-line treatment in Peru. Pentavalent antimony could then be used only in those cases that do not respond to the combination topical therapy.

## Supporting Information

Checklist S1 CONSORT checklist.
(0.12 MB PDF)

Protocol S1 Trial protocol.
(0.29 MB PDF)
