# Supplementary material for: First-Line Therapy for Human Cutaneous Leishmaniasis in Peru Using the TLR7 Agonist Imiquimod in Combination with Pentavalent Antimony
Source: PLoS Negl Trop Dis. 2009 Jul 28;3(7):e491. doi: 10.1371/journal.pntd.0000491 (PMC2710502; doi:10.1371/journal.pntd.0000491)
Supplement: Protocol S1 — Trial protocol. (0.29 MB PDF) [file pntd.0000491.s002.pdf]

Title: Randomized double blind clinical trial of imiquimod (Aldara) versus placebo used in combination with pentavalent antimony (Glucantime) in Peruvian cutaneous leishmaniasis patients.

Principal Investigators

Dr. Greg Matlashewski[1]

Dr. Alejandro Llanos-Cuentas[2]

Dr. Brian Ward[1]

Dr. Theresa Gyorkos[1]

Co-investigators

Dr. Cesar Miranda-Verastegui[1,2]

Dr. GianFranco Tulliano [2]

Dr. Maria Cruz[2,3]

Dr. Wessmark Calderon[2]

Statistician

Dr. Elham Rahme[1]

[1]McGill University, Montreal, Canada

[2]Universidad Peruana Cayetano Heredia, Lima, Peru

[3]Social Security Hospital ESSALUD-Cusco

Sponsor

Drugs for Neglected Diseases Initiative, Geneva, Switzerland (DNDi)

Signature Page

**Principal Investigators**	**Signature**	**Date**
Dr. Greg Matlashewski		
Dr. Alejandro Llanos-Cuentas		
Dr. Brian Ward		
Dr. Theresa Gyorkos		
**Co-investigators**		
Dr. Cesar Miranda-Verastegui		
Dr. Gianfranco Tulliano		
Dr. Maria Cruz		
Dr. Wessmark Calderon		
**Statistician**		
Dr. Elham Rahme		

Table of Contents

		Page
1. Introduction		5
2. Trial objective		6
3. Trial design		6
4. Eligibility criteria		
4.1 Inclusion criteria		8-9
4.2 Exclusion criteria		9
5. Screening evaluations		9
6. Randomization, blinding and assignment of treatment		11
7. Treatment:		
7.1	Antimonial treatment	12
7.2	Imiquimod or placebo treatment	12
8. Evaluation of clinical response:		
8.1	Criteria to evaluate the efficacy of the treatment (efficacy)	13
8.2	Baseline (Day 1): evaluation and treatment procedures	14
8.3.	Days 2-9 evaluation and treatment procedures	15
8.4	Day 10 evaluation and treatment procedures	16
8.5	Days 11-19 evaluation and treatment procedures	17
8.6	Day 20 (Last day of treatment) evaluation and treatment procedures	17

8.7 Analysis of the response to therapy at 1, 2, 3, 6, 9 and 12 months after the therapy 18

8.8 Safety and tolerability evaluation

8.8.1 Grading of side effects of the treatment at the cream application site 18

8.8.2 Grading of pain at the cream application site 19

8.8.3 Grading of pruritus (itching) at the cream application site 19

8.8.4 Grading of erythema (inflammation) at the cream application site 19

8.8.5 Grading of swelling at the cream application site 20

9. Follow-up Evaluations 20

10. Adverse event management 21

11. Patient removal from the study 24

12. Concomitant Medications 24

13. Treatment preparation 25

14. Handling, Shipping , Storage 25

15. Statistical Considerations 26

16. Administrative Procedures 27

17. Relevant References 30

1. Introduction.

Current treatment of cutaneous leishmaniasis is inadequate, toxic, costly, requires repeated injections, and is not effective in all cases. Pentavalent antimonials were developed over a half century ago, and are administered by repeated injection for 20 consecutive days. They are expensive and toxic (fatigue, cardiopathy, pancreatitis), yet they remain the mainstay of anti-*Leishmania* therapy in the developing world. New paradigms to treat this chronic infection are needed. .

Strong rationale for this proposal emanates from the integration of distinct and important recent developments. First, a relatively new immunomodulating macrophage activating compound, termed imiquimod (Aldara^{TM}) has been developed by 3M Pharmaceuticals and has regulatory approval in the US, Canada, and the European Union for the treatment of skin infections with human papillomaviruses (HPV). As a topical agent, imiquimod has been shown to be safe and effective against HPV infections. Therefore the cost of development has already been incurred. Second, we have shown that imiquimod can activate macrophage killing of *Leishmania in vitro* and in mouse model experiments. Third, we have completed phase I clinical trials in Peru showing that, in cutaneous leishmaniasis patients which have not responded to treatment with a standard course of pentavalent antimony (Glucantime), the combination of imiquimod with standard pentavalent antimony therapy was safe and significantly increased the rate of cure with *L. (V) braziliensis* and *L. (V) peruviana* infections (Clin. Infect. Dis. 33: 1847-1851, 2001; Clin. Infect. Dis. 40: 1395-1403, 2005). These developments provide a foundation to establish whether cutaneous leishmaniasis patients in Peru who have not previously been treated with pentavalent antimony can be treated with a combination of pentavalent antimony (Glucantime^{TM}) and imiquimod (Aldara^{TM}). This study will be conducted in accordance with ICH harmonised tripartite guidelines for good clinical practice.

2. Trial Objective:

The objective is to determine whether patients who have not previously been treated with pentavalent antimony can be more effectively treated with a combination of pentavalent antimony and imiquimod than with pentavalent antimony alone.

This study will test whether addition of imiquimod to standard antimony therapy provides a significant benefit in subjects with newly diagnosed disease. Based on our previous results, we hypothesize that lesions in patients who receive the combined treatment of pentavalent antimony and imiquimod as a first line therapy will resolve more rapidly and produce less scarring than treatment with pentavalent antimony alone. This is a relevant question since our previous studies involved only patients with cutaneous leishmaniasis who had previously failed one course of pentavalent antimony therapy (Clin. Infect. Dis. 33: 1847, 2001; Clin. Infect. Dis. 40: 1395, 2005).

3. Trial Design

A randomized, double-blind clinical trial will involve a total of 80 patients. The randomization procedure is detailed below in section 6 under Randomization, blinding and assignment of treatment. The trial will be conducted at the Instituto de Medicina Tropical Alexander von Humbolt -Universidad Peruana Cayetano Heredia (IMTAvH-UPCH) in Lima site for patients infected with *L. peruviana* and the IMTAvH-UPCH Cusco site, for patients infected with *L. braziliensis*. The groups will be assigned the following regimens: The Lima arm will include 20 experimental and 20 placebo patients. Likewise, the Cusco arm will include 20 experimental and 20 placebo patients.

<u>Control</u>: Standard dose of pentavalent antimony (Glucantime™ 20 mg/kg IV infusion/day for 20 days) plus placebo cream (thin layer cream applied to each lesion three times per week for 20 days).
<u>Experimental:</u> Standard dose of pentavalent antimony (Glucantime™ 20 mg/kg IV infusion/day for 20 days) plus standard dose of Aldara™ (5% imiquimod cream applied

to each lesion three times per week for 20 days). All patients will be subjected to follow-up clinical evaluations at 1, 2, 3, 6, 9 and 12 months after the last treatment (Day 20). The follow up evaluations will be performed by the investigation team. The endpoint for clinical cure will be complete re-epithelialization of the lesion with no sign of inflammation.

Patients will be recruited from highly endemic areas which are within one day driving from the study centers in Lima and Cusco including Ancash, Churin, Yumpe, , Sicuani, and surrounding areas. Note, that patients treated in Cusco have predominantly *L. braziliensis* infections and patients treated in Lima, Ancash, Churin and Yumpe have predominantly *L. peruviana* infections. Study teams (consisting of a UPCH trained doctors, nurses, and technicians) will carry out the treatments at IMTAvH Lima site under the supervision of Dr. Gianfranco Tulliano and at IMTAvH Cusco site under the supervision of Dr. Maria Cruz. Dr. Alejandro Llanos will oversee the treatments and follow-up at each center. Following the completion of therapy, the teams will then coordinate follow-up visits at 1, 2, 3, 6, 9 and 12 months. Dr. Cesar Miranda will coordinate the follow up visits as he has done previously in these types of studies.

Personnel Responsibilities in Peru

Two research units will be established to work out at the IMTAvH –UPCH Lima and Cusco sites. This will ensure sufficient patients are recruited at each site and also determine whether the treatments work similarly for *L. braziliensis* (Cusco site) and *L. peruviana* (Lima site). All personnel will have been trained by Dr. Alejandro Llanos-Cuentas at UPCH.

Lima Site

This will be the study coordination site where the Peruvian Principal Investigator Dr Alejandro Llanos-Cuentas will be based and from where the project will be managed. He will be supported by an administrative assistant, an accountant and the study pharmacist. The Lima site study team consists of an MD, study nurse, laboratory technician and field

worker who will recruit and care for the patients during the study and carry out all study procedures. The Lima site will be the place for the study coordinator, who will work together with Dr Alejandro Llanos-Cuentas.

Cusco Site

A team consisting of 2 MDs, study nurse and field worker will staff this trial site and will be supported by the Lima group

Samples will be sent to UPCH in Lima to for PCR analysis, culturing of parasites and storage.

4. Eligibility Criteria

4.1 Inclusion Criteria

Patients must meet ALL of the following criteria to be eligible for inclusion into the study.

a) Males and females between the ages of 5 and 65 years
b) Confirmed diagnosis of cutaneous leishmaniasis (CL) defined as:
   * Presence of an active ulcerative cutaneous *Leishmania* lesion, and
   * Positive identification of parasite from lesion smear (e.g.: microscopy, culture, or PCR)
c) Time of disease more than 4 weeks.
d) No prior therapy for CL with anti-leishmanial agents.
e) Female patients of childbearing potential must have a negative urine pregnancy test at screening, must not be breast-feeding and are required to use adequate contraception during the 20 days of the treatment.
f) Must give informed written consent or have a parent provide consent for anyone under the age of 18. The patient must be willing to participate in all treatment evaluation visits, be reachable by telephone or accessible to personal contact by the study site personnel.

g) Stability of residence or be reachable by the study personal.

4.2 Exclusion Criteria

Patients must have NONE of the following criteria to be eligible for inclusion into the study:

a) Having a lesion(s) > 25 cm^2
b) More than 6 cutaneous lesions.
c) Have a mucosal lesion.
d) History of previous exposure to imiquimod or anti-*Leishmania* treatment.
d) Participation in another experimental protocol and/or have received investigational products within 30 days prior to study entry.
e) History of any acute or chronic illness (other than CL) or medication that, in the opinion of the investigators, may interfere with the evaluation of the trial (i.e History of heart illness, corticotherapy)
f) History of significant psychiatric illness.
g) History of previous anaphylaxis or severe allergic reaction to one or more of the proposed drugs.
h) Patients who are unlikely to cooperate with the requirements of the study protocol.
i) Concomitant infection (ex. Bartonellosis, Sporotrichosis, Mycobacteria)
j) Women who are pregnant or breast feeding.

..

These inclusion and exclusion criteria were established in order to provide the optimal opportunity for treatment response.

5. Screening Evaluations

Screening evaluations will occur within the 30-day period prior to Day 1 (i.e. Day –30 to Day 0). The purpose of the screening period is to ensure that the patients meet

all eligibility criteria and that they entirely comprehend the protocol and its requirements before signing the consent form and agreeing to enter the trial.

The screening procedure includes the following assessments as listed below and recorded on the **clinical report form (CRF)** for the study:

a) Provision of study informed consents and patient signature of these informed consent documents.

b) Positive diagnosis of CL by smear or PCR. PCR will be used to verify whether the patient is infected with *L. braziliensis* or *L. peruviana*

c) Urine pregnancy test for any female patients of childbearing potential. Test result must be negative in order to proceed.

d) Documentation of medical history.

e) Record of any concomitant medications.

f) Assessment of vital signs.

g) Perform a <u>complete</u> physical exam. The location, size and description of the active cutaneous lesions must be noted. Every mucosal membrane will be examined and record the presence or absence of any mucosal lesion.

h) Record any other disease symptoms the patient may experience.

i) Electrocardiogram: Will be performed prior to treatment

j) Collect blood as follows:

   Approximately 2.5 ml anticoagulated (EDTA) whole blood for CBC, white cell differential and platelet count.

k) Approximately 8.5 ml whole blood into serum separator tube (SST) for serum chemistry and serology testing for chemistries to include: ALT, AST, total bilirubin, alkaline phosphatase, pancreatic amylase, glucose and creatinine.

l) Obtain skin biopsy for diagnostic procedures (except for lesions from the face).

6. Randomization, blinding and assignment of treatment.

A randomization list from 1-80 will be generated by Dr. Elham Rahme at McGill University using a ratio of 1:1 to either the pentavalent antimony plus imiquimod arm or the pentavalent antimony plus placebo arm. The list will then be provided to a pharmacist, Ana Graña at UPCH who will be responsible for therapy preparation. This individual will not be involved in conducting the trials and therefore cannot administer the treatment or conduct patient evaluation(s).This list will contain the study I.D. numbers (1-80) and corresponding treatment assignments and remain completely blinded to the study investigators who will be treating and evaluating the patients.

Each patient will be assigned by the study doctor a study I.D. number from the above randomized list assigned in sequential order when he/she is enrolled in the trial. The study doctor at each site will ask to the study coordinator for the study ID number. The study ID number will be provide to the study coordinator by the pharmacist. This study ID number will be entered on the CRF for that patient. The nurse will then administer the treatment as detailed in section 7. The study doctor will carry out the evaluation and the nurse, doctor, and technician will remain blinded for the treatment and evaluation period.

7. Treatment

The randomized treatment assignment will take place on Day 1. In the case of bacterial superinfection, patients will receive a complete course of oral or systemic antibiotic treatment (i.e., dicloxacillyn or clindamycin) to clear the infection before entry into the trial (during the screening period).

The nurse will record the drug administration of each patient in the CRF and the doctor will record adverse effects and clinical outcomes in the CFR.

7.1 Antimonial treatment:

The total dose of pentavalent antimony (20 mg Glucantime™ /kg body weight/per day) for 20 consecutive days will be administrated by intravenous with a syringe gauge # 23. Injections will be given by the nurse in alternating sites (i.e. veins in upper arms). Because a total of 20 injections will be administered during the course of the study, care must be taken to ensure that injections are not administered in the same exact location.

7.2 Imiquimod and placebo treatment:

On Day 1 of treatment, the treatment package containing the assigned therapy for an individual patient will be opened and the sachet containing imiquimod or placebo cream will be applied to the cutaneous lesion. Wearing gloves, the nurse will clean the lesion using saline solution removing debris or scab. The nurse will apply the cream on the entire area of each lesion (including a 0.5 cm margin of normal skin). The cream will be applied in the morning and will be rubbed into the lesion(s) until no longer visible. Each sachet will contain approximately 250 mg of cream and the lesion will receive between 125–250 mg of cream. The topical treatment will be applied three times per week (p.e. Monday, Wednesday, Friday or Tuesday, Thrusday and Saturday) , in total 9 applications concurrent with the 20 day course of treatment with pentavalent antimony. Only new unopened sachets provided by 3M pharmaceuticals will be used for each treatment.

The lesion will remain uncovered for at least 30 minutes following application during which time the study doctor will record all symptoms of the patient. It will not be necessary to use occlusive dressings for lesions on the face. However, for the lesions not on the face, the lesion(s) will be covered to prevent cream removal by clothing. The patients will be instructed to remove the cover and wash the lesions approximately 8 h following treatment and each following morning.

After each treatment, the study doctor will evaluate the patient for 30 minutes. In the event of an acute reaction, appropriate medical care will be provided by the

physician as appropriate. After the 30-minute observation period, the patient can continue to do his/her daily activities and return to the clinic on the following day.

8. Evaluation of Clinical Response

The clinical response will be evaluated using standard descriptive criteria for assessing the size and severity of the cutaneous lesion. This will include, but will not be limited to: size of lesion, reduced ulceration, re-epithelialization, and inflammation reduction. The evaluation will be recorded by the study doctor and recorded on the **clinical report form (CRF).**

8.1 Criteria to evaluate the response to treatment (efficacy)

A standard scale will be used:

Stage	Observations
M0	No improvement. The lesion is active and has the same characteristics or has become larger than before the start of treatment.
M1	The size of the lesion is decreased ~50% in comparison with the initial lesion. Less inflammatory signs with discrete re-epithelialization.
M2	The size of the lesion is decreased between 50-90% in comparison with the initial lesion. Few inflammatory signs, less than M1.
M3	The size of the lesion is decreased more than 90% with re-epithelialization and very little inflammation.

M4	Complete re-epithelialization with a characteristic scar and no inflammation.

8.2 Baseline (Day 1): Evaluation and treatment procedures.

Patients who meet all inclusion/exclusion criteria at the screening visit will be asked to return to the clinic for the baseline visit (Day 1). Upon arrival, the following evaluations will be completed and recorded on the CRF by the study doctor:

8.2.1 Study I.D. number will be assigned by the pharmacist to the patient and placed on the CFR and consent form by the study doctor at each site .

8.2.2 Vital signs assessment. Patients with abnormal pulse rate, blood pressure or oral temperature >38°C (100.4°F) or having acute illness on the day of the therapy must NOT receive the therapy. Temperature and acute illness must resolve within 7 days of initial scheduled visit in order to proceed with the study.

8.2.3 Limited physical exam, noting any changes from the screening examination. This must include any changes in the cutaneous lesion description obtained during the screening visit. Note: Do NOT proceed if patient has developed a mucosal lesion.

8.2.4 Female patients of childbearing potential must have a documented negative urine pregnancy test on day 1 of therapy and the date of the last menstrual period recorded.

8.2.5 Record any concomitant medications.

8.2.6 Make a drawing of each lesion using a plastic sheet, drawing the borders of each lesion on Day 1, and place in the CRF.

8.2.7 Photograph all cutaneous lesions using a digital camera with daylight (no flash) and with a distance of 15 cm from the center of lesions. A standard rule with a label of the ID patient code and date will be placed beside the lesion before taking the photograph. The pictures will be stored in a computer at IMTAvH sites and they will be accessed only by the study team.

Upon completion of these evaluations, the study doctor will review the inclusion/exclusion criteria for each patient. Patients who meet all eligibility criteria will have the following additional procedures completed on Day 1:

8.2.8 Provide the treatment (study nurse) as described in Section 7.

8.2.9 Monitor adverse events and cream application site reactions for 30 minutes post treatment and document on the CRF.

8.3 Days 2-9 evaluation and treatment procedures

8.3.1 Record any concomitant medications on the CRF.

8.3.2 Perform limited physical examination (study doctor).

8.3.3 Assess vital signs prior to treatment application.

8.3.4 Assess the active cutaneous lesions identified during the screening/baseline visits. Note presence and description of any additional lesions or mucosal lesions that may have appeared since the previous visit.

8.3.5 The study nurse will administer the treatment to the patient as described in Section 7 as long as there are no severe adverse events as detailed in section 10.1.

8.3.6 Monitor adverse events and injection site reactions for 30 minutes post treatment and document on the CRF.

8.4 Day 10 evaluation and treatment procedures

8.4.1 Record concomitant medications on the CRF.

8.4.2 Perform a complete physical examination (study doctor).

8.4.3 Assess vital signs prior to treatment application.

8.4.4 Assess the active cutaneous lesions identified during the screening/baseline visit. Note presence and description of any additional lesions that may have appeared since previous visit. Stop therapy if a mucosal lesion has developed

8.4.5 Record adverse events occurring since the last evaluation.

8.4.6 Take a drawing of each lesion using a plastic sheet, drawing the borders of each lesion.

8.4.7 Obtain photograph of the representative cutaneous lesion first photographed at baseline following the same procedures of section 8.2.7

8.4.8 Collect blood for safety evaluations as follows (study technician): Approximately 2.5 ml anticoagulated (EDTA) whole blood for CBC, white cell differential and platelet count.

Approximately 8.5 ml whole blood into serum separator tube (SST) for serum chemistry testing. Chemistries to include: ALT, AST, total bilirubin, alkaline phosphatase, pancreatic amylase, glucose and creatinine.

8.4.9 Electrocardiogram: Only if a cardiovascular condition should be monitored.

8.4.10 Administer treatment to the patient as described in Section 7 as long as there are no severe adverse events as detailed in section 10.1 .

8.5 Days 11-19 evaluation and treatment procedures

8.5.1 Record any concomitant medications.

8.5.2 Perform limited physical examination.

8.5.3 Assess vital signs prior to treatment application.

8.5.4 Assess the active cutaneous lesions identified during the screening/baseline visit. Note presence and description of any additional lesions or mucosal lesions that may have appeared since previous visit.

8.5.5 Administer treatment to the patient as described in Section 7 as long as there are no severe adverse events as detailed in section 10.1.

8.5.6 Monitor adverse events and injection site reactions for 30 minutes post treatment and document on the CRF.

8.6 Day 20 (Last day of treatment) evaluation and treatment procedures

8.6.1 Record concomitant medications.

8.6.2 Perform a complete physical examination.

8.6.3 Assess the active cutaneous lesions identified during the screening/baseline visit. Note presence and description of any additional lesions that may have appeared since previous visit.

8.6.4 Record adverse events occurring since the last evaluation.

8.6.5 Obtain photograph of the representative cutaneous lesion first photographed at baseline following the same procedures of section 8.2.7

8.6.6 Collect blood for safety evaluations as follows:

Approximately 2.5 ml anticoagulated (EDTA) whole blood for CBC, white cell differential and platelet count.

Approximately 8.5 ml whole blood into serum separator tube (SST) for serum chemistry testing. Chemistries to include: ALT, AST, total bilirubin, alkaline phosphatase, pancreatic amylase and creatinine.

8.6.7 Electrocardiogram: Only if a cardiovascular condition should be monitored during the antimony therapy.

8.6.8 Administer treatment to the patient as described in Section 7 as long as there are no severe adverse events as detailed in section 10.1.

Patients will be evaluated during the follow up period by the study team as described in the section 9.

8.7 Analysis of the response to therapy at 1, 2, 3, 6, 9, 12 months of follow-up:

Improvement: Significant reduction in the size of the lesion at the time of evaluation (Stages M1-M3) compared to baseline.

Cure: Complete re-epithelialization of the lesion without inflammation (Stage M4).

Failure: Any one of the following or combined options

Stage MO at 1 month of follow up after the treatment
Stage M1 at 3 months of follow up after the treatment

Regression of the clinical stage to a lower stage or development of a new lesion.

8.8 Safety and tolerability evaluation

8.8.1 Grading of side effects of the treatment at the at the cream application site (pain, pruritus, erythema and swelling):

Grade 0 = none

Grade 1 = mild (easily tolerated)

Grade 2 = moderate (sufficiently discomforting to interfere with daily activities)

Grade 3 = severe (prevents normal daily activity)

8.8.2 **Grading of pain at the site of application site** will be according to the following definitions:

None

Mild (Some pain but does not limit the movement of the place where the lesion is located.)

Moderate (Limits movement)

Severe (Cannot move the place where the lesion is located)

8.8.3 **Grading of pruritus (itching) at the cream application site** will be made according to the following definitions:

None

Mild	(Noticeable but easily tolerated)
Moderate	(Sufficiently discomforting to slightly interfere with daily activities)
Severe	(Constant discomfort. Leads to restriction of normal daily activities)

8.8.4 **Grading of erythema (inflammation) at the cream application site** will be according to the following:

None	(0 to <1 cm in diameter)

Mild	(1 to 4 cm in diameter)
Moderate	(5 to 9 cm in diameter)
Severe	(10 cm or greater in diameter)

8.8.5 **Grading of swelling at the cream application site** will be according to the following:

None	
Mild	(Some tenderness)
Moderate	(Limits movement of the place where the lesion is located)
Severe	(Cannot move the place where the lesion is located)

9. Follow-up Evaluations

All patients will attend follow-up clinical evaluations at 1, 2, 3, 6, 9 and 12 months after the last treatment (Day 20). The follow up evaluations will be performed by the investigation team and will include the following procedures:

9.1 Record all evaluations on the CRF: disease status based on the less improved lesion (worse stage) in the case of a patient with more than one lesion. Registration of any additional therapy for leishmaniasis that the patient may have received. Take special note of the lesion size, inflammation, and re-epithelialization and score lesion evolution as detailed in section 8. Note presence and description of any additional lesions or mucosal lesions that may have appeared since previous visit.

9.2 Obtain photograph of the representative cutaneous lesion first photographed at baseline as described in section 8.2.7

9.3 Perform limited physical examination.

9.4 Record all adverse events since the last study visit.

10. Adverse Event Management.

10.1 Adverse events

An adverse event will be defined as any noxious, pathological or unintended change in anatomical, physiological or metabolic functions as indicated by physical signs, symptoms and/or laboratory changes occurring in any phase of the clinical trial, whether or not they are considered to be associated with the study drug. This includes an exacerbation of pre-existing conditions or events, intercurrent illnesses, drug interaction, or the significant worsening of the disease under investigation. Anticipated day to day fluctuations of pre-existing conditions, including the disease under study, that does not represent a clinically significant exacerbation or worsening of the condition, will not be considered adverse events.

All adverse events occurring after the start of the study (defined as when informed consent was obtained) are to be reported. This is regardless of whether or not they are considered to be drug-related. Adverse events (AEs) will be elicited by the

investigator asking the patient or the patient's parent or guardian a non-leading question such as "Do you/has your child felt different in any way since starting the new treatment or since the last assessment?" If the response is "Yes", the nature of the event, the date and time (where appropriate) of onset, the duration, maximum intensity (see below) and relationship to treatment are to be established (see below). Details of any changes to the dosage schedule or any corrective treatment are to be recorded on the appropriate pages of the CRF.

Assessment of Intensity/Severity

The assessment of intensity/severity will be based on the investigator's clinical judgment and categorized as follows:

Mild: An adverse event which is easily tolerated by the patient, causing minimal discomfort and not interfering with daily activities.

Moderate: An adverse event, which is sufficiently discomforting to interfere with normal daily activities.

Severe: An adverse event, which prevents normal daily activities.

Assessment of Causality

The investigator will use clinical judgment to determine the degree of certainty with which an adverse event is attributed to drug treatment. Alternative causes, such as natural history of the underlying diseases, concomitant therapy, etc are to be considered taking into account the known pharmacology of the drug, any previous reactions, literature reports and relationship to time of drug ingestion. Causality will be assessed using the following categories: not related, unlikely,

suspected (reasonable possibility) or probable. Patients with adverse events will be followed-up until the event disappears or the condition stabilizes.

If the treatment becomes intolerable to the patient for any reason (eg. Inflammatory response) this should be recorded as an AE and the cream application stopped.

Serious Adverse Events

A serious adverse event will be defined as any event which is fatal, life threatening, disabling or incapacitating or results in hospitalization, prolonged hospital stay or is associated with congenital abnormality, cancer or overdose (either accidental or intentional). In addition, any experience which the investigator regards as serious or which suggests any significant hazard, contraindication, side effect or precaution that might be associated with the use of the drug will be reported as a serious event. Any serious adverse event occurring either during the study or within 30 days, of receiving the last dose of study medication, is to be reported by telephone to the study monitor within 24 hours. This will be followed by a full written summary containing copies of relevant hospital case records and autopsy reports where applicable.

As treatment is by topical application, over dosage is not anticipated. However, in the event of over dosage (error of dosage calculation or administration), this will be communicated to the Principal Investigator in Peru, Dr. Alejandro Llanos, within 24 hours or as soon as possible thereafter. Details of any signs or symptoms and their management will be recorded on the CRF including details of any antidote(s) administered. As there are no specific antidotes available for the medications to be used in this study, patients will receive all supportive care needed at discretion of the treating physician and after consultation with the study coordinator above.

In a case of a *Serious Adverse Event*, the treatment code has to be opened by the investigators.

11. Patient Removal from Study

Patients will be free to withdraw from the study at any time, for any reason, and without prejudice to further treatment. If the treating doctor determines that the clinical situation is worsening to an extent greater than a) one would expect from the natural history of the disease, or b) the range of clinical progression seen in previous cohorts of patients with similar initial clinical findings, he/she may elect to remove the patient from the clinical trial and to initiate treatment with the best medical regimen available.

Patients may also be removed from the study by the investigators, if necessary, in the event of a severe adverse event, pregnancy, or if the patient develops a concurrent illness which would preclude continuation in the study. Patients who are withdrawn from the study due to concurrent illness will be counseled by the investigators to receive the appropriate care. Patients who are removed from the study will undergo medical evaluations and will be followed for toxicity as closely as possible. Any patient who discontinues, regardless of the reason, will not be allowed to re-enroll in this study. The CRF will include reasons for withdrawal and any necessary treatment. Adverse events will be reported by the investigators and to the Ethics committee at UPCH and McGill University.

12. Concomitant Medications

The use of any medication will not result in discontinuation of the patient from the study unless the medication(s) will interfere with the interpretation of the results of this study or will be unsafe for the patient. All medications, whether prescription or non-prescription, taken from the day of the first treatment (Day 1) through the end of the study (Month 12 of follow up) must be recorded on the CRF.

13. Treatment preparation

Packaging

The pharmcist, Ana Graña at UPCH will be responsible for preparing the treatment packages containing either the placebo or imiquimod cream. She will provide the study packages to the doctor/nurse who will be carrying out the trials. Each package will contain the treatment indicated in the randomization list that will be generated by Dr Elhamn Rahme the unblinded statistician at McGill University. Each package will contain the treatment consisting of either placebo or imiquimod cream (40 sachets in each package) clearly labeled with the study I.D. number. Each package will be properly sealed and no evidence other than the study I.D. number will be indicated on each package.

14. Handling, Shipping, Storage

Investigational Supplies and Handling/Preparation

The Aldara (Imiquimod 5% cream sachets) and the identical in appearance placebo cream will be provided by 3M Pharmaceuticals. Diagnosis and treatment supplies will be provided in kits assembled at IMTAvH-UPCH Lima site which will contain gloves, gauze, cotton, slides, scalpels, single-use syringes, needles, culture tubes, antibiotics, etc..

The Aldara and placebo creams will be shipped under refrigerated conditions and will be stored upon arrival at the site in a monitored refrigerator maintained at 2-8ºC in a secure, controlled location. All investigational study supplies will be stored in a locked, safe area to prevent unauthorized access. Long term storage of Aldara and placebo will be at 2-8ºC, but these can be transported and used at room temperature.

Receipt of Supplies

The pharmacist is responsible for making an inventory of each shipment of investigational supplies received, and writing this into an accountability form. Accurate accounting of all investigational material will be made. Unused imiquimod or placebo must be retained for drug accountability by the trial Monitor.

15. Statistical Considerations

The randomized clinical trial study design uses a rigorous methodology to evaluate the clinical research questions posed in this proposal. The sample size has been calculated based on estimates from previous published data results and will ensure adequate statistical power while maintaining efficiency. Minimal loss to follow-up is expected (<10%). The primary analyses will be conducted excluding patients with missing outcomes. Intention-to-treat analysis will also be performed (ie. including all patients randomized).

Descriptive statistics (means ± standard deviation (SD), proportions and, when required, medians and interquartile ranges) will be performed to compare socio-demographic, clinical and epidemiological characteristics of patients at baseline between experimental and control groups. These characteristics include: age, sex, region of residency and education/occupation, among others. Proportions of patients cured at each of the follow up dates will be assessed. Proportions will be compared between the two groups using chi-square tests. Means derived from continuous variables will be compared using t tests or non-parametric tests, as appropriate.

Time-to-cure will be compared between the two groups using Kaplan-Meier curves and crude rates of cure will be calculated by person-years. For comparison purposes, we will assume that cure occurred at the time of the visit for cured patients. In a randomized clinical trial, we expect to find no differences in patient characteristics

between the two groups. However, when sample sizes are small, it will likely be necessary to adjust for slight imbalances in independent variables between the two groups. Cox proportional-hazard models will be constructed to determine the effect of treatment on time-to-cure, adjusting for patients' baseline characteristics. Results will be expressed in terms of hazard ratios and 95% confidence intervals. The proportional hazard assumption will be examined using graphical methods and the proportional hazard test in SAS PHGLM. Secondary analyses will be conducted in a similar manner, assuming that all those who were lost to follow-up were cured only at the end of the 12 month follow-up period.

Assuming a constant hazard ratio over time, with a sample size of 40 subjects in each of the two intervention groups (total of 80 subjects), the log-rank test for equality of survival curves will have 80% power to detect a statistically significant difference in proportions cured between the two groups based on a difference in cure proportions of at least 32% (hazard ratio of 2.6, proportion cured in the control group of 35% and proportion cured in the treated group of 67% - (less than the 72% observed at 3 months in our previous trial). If the proportion cured in the experimental group is higher than 67%, then this will increase the statistical power of the test. We believe that this will provide sufficient and compelling evidence for policy-makers.

16. Administrative Procedures

16.1 Ethics Review Board Approval

The experimental protocol for this study has been designed in accordance with the general ethical principles outlined in the Declaration of Helsinki, 2000 and ICH guidelines for Good Clinical Practice(International Committee for Harmonisation) This protocol will be reviewed by the Ethics Review Board (ERB) of UPCH, McGill University, The National Institute of Health in Peru (INS-Peru), and the local IRB for areas where the patients will be treated.

The investigators will be responsible for preparing documents for submission to government and ERBs and obtaining written approval for this study. All approvals will be obtained prior to the initiation of the study.

16.2 Informed Consent

The informed consent form will be in Spanish, the patient's native language (Appendix 2). The Investigator or his/her staff will explain the nature of the investigation and the risks involved to each patient prior to enrollment, and will obtain written informed consent. The patient will also be informed that he/she is free to voluntarily withdraw from the study at any time. The patients will be provided with an information sheet which will summarize the nature of the trial.

16.3 Study Monitoring

The investigators will allow representatives of DNDi and the study monitor to periodically monitor the study, all CRFs and corresponding source documents for each patient will be made available. It is important that the investigators and other trial site staff are available at these visits. The monitoring visits provide DNDi with the opportunity to evaluate the progress of the study, verify the accuracy and completeness of CRFs, resolve any inconsistencies in the study records, as well as to ensure that all protocol requirements, applicable regulations, and investigator's obligations are being fulfilled. The investigators must maintain source documents such as laboratory and consultation reports, history and physical examination reports, etc., for possible review. The Principal Investigator will record the date of each visit together with a summary of the status and progress of the study. Proposed actions will be confirmed in writing. The investigators will allow audit of the trial by any external regulatory agency to verify data submitted.

16.4 Modification of Protocol

No deviations from the protocol may be made. The investigators may approve minor exceptions on a case-by-case basis. If modification of the protocol is necessary, the modification must be initiated and confirmed in writing by the investigators and approved by the ERB prior to implementation.

16.5 Departure from Protocol

If an emergency occurs that requires a departure from the protocol for an individual, the departure will only be for that individual. The physician in attendance in such a situation will, if circumstances and time permit, contact the Principal Investigators (Dr Llanos Cuentas in Lima and Dr Matlashewski in Montreal) immediately by telephone. The CRF will detail the departure from the protocol and state the reasons for such departure. ERBs must be notified immediately if the departure from the protocol affects the safety or rights of the patient.

16.6 Suspension of Study

If safety concerns arise during the study, the ERBs may recommend to the investigators that the study be suspended, amended or terminated. The study may be suspended until the situation or safety concern has been resolved.

16.7 Use of Information and Publications

All information contained in this clinical study protocol, and accompanying documents, is confidential and considered the property of DNDi, UPCH, McGill University. The investigators agree to use this information and data only in accomplishing this study and will not use it for other purposes without permission of DNDi, UPCH, McGill University,. DNDi, UPCH and McGill University encourage publication in peer-reviewed medical journals and will not unduly withhold

permission to publish. All proposed publications, papers, abstracts or written materials related to the study or an outline of any oral presentation, shall be submitted to DNDi, UPCH, and McGill University for approval.

16.8 Record Retention

The investigators shall retain the clinical study records until such time as directed by DNDi , UPCH and McGill University. Records are to be retained for at least 10 years after regulatory approval or if not approved, until 10 years following the completion of the trial including follow-up.

16.9 Study Insurance

Insurance for the study will be purchased. The maximum limit of indemnity per person insured shall be $1,000,000. This insurance can be purchased from for example HDI, Munchen, Germany. However, the insurance company to be used for this study has yet to be identified.

17. Relevant References

Miranda-Verástegui, C., Arévalo, I., Llanos-Cuentas, A., Ward, B., and Matlashewski, G. Randomized, double blind clinical trial of topical treatment 5% imiquimod (Aldara) with parental meglumine antimonate (Glucantime™) in the treatment of cutaneous leishmaniasis in Peru. *Clin. Infect. Dis. 40: 1395-1403, 2005*

Arevalo, I., Ward, B., Miller, R., Meng, TC, Najar, E., Alvarez, E., Matlashewski, G., and Llanos-Cuentas, A. Successful treatment of drug-resistant cutaneous leishmaniasis in humans by using imiquimod, an immunomodulator. *Clin. Infect. Dis. 33: 1847-1851, 2001.*

Buates, S., and Matlashewski, G. Treatment of experimental leishmaniasis with the immunomodulators, imiquimod and S-28463: efficacy and mode of action. *J. Infect. Dis. 179: 1485-1494, 2001*
